# Sepsis in PD-1 light

**DOI:** 10.1186/s13054-016-1370-x

**Published:** 2016-07-05

**Authors:** Guillaume Monneret, Morgane Gossez, Fabienne Venet

**Affiliations:** Hospices Civils de Lyon, Immunology Laboratory, Hôpital E. Herriot, 5, place d’Arsonval, 69437, Lyon, Cedex 03 France; EA 7426 PI3 (Pathophysiology of Injury-induced Immunosuppression), Hospices Civils de Lyon/Université Claude Bernard Lyon 1/bioMérieux, Hôpital E. Herriot, 5, place d’Arsonval, 69437, Lyon, Cedex 03 France

## Abstract

Increasing evidence suggests that after the first pro-inflammatory hours, sepsis is characterized by the occurrence of severe immunosuppression. Several mechanisms have been reported to participate in sepsis-induced immune alterations affecting both innate and adaptive immunity. Of these, the concept of ‘cell exhaustion’ has gained a lot of interest because some parallels can be drawn with the cancer field in which immunostimulation approaches through blocking immune checkpoints currently obtain remarkable success. Herein, perspectives regarding co-inhibitory receptors’ contribution to lymphocyte exhaustion in sepsis will be discussed in the context of a recently published study investigating the potential of PD-1 molecule expression (i.e. PD-1 on lymphocytes, PD-L1 on monocytes) to predict mortality in septic shock patients.

The work by Shao et al. [1] provides further insights regarding the potential of Programmed Death-1 (PD-1)-related molecule expression to predict mortality in septic shock patients. These immune checkpoint molecules constitute a system of negative regulators involved in controlling T-cell responses. Co-ligation of TCR with PD-1 molecules induces an inhibitory signal in T cells characterized by cell cycle arrest, inability to proliferate and reduced cytokine synthesis (IFN-γ and/or IL-2). This system is composed of PD-1 (CD279) and its two ligands, PD-L1 (B7-H1, CD274) and PD-L2 (B7-DC, CD273). Viruses and tumour cells take advantage of this pathway to escape the host’s immune defences. In sepsis, pioneering work by Pr Ayala’s group published in 2009 reported that PD-1 knockout mice exhibited greater capacity to clear bacteria and lower mortality after experimental sepsis [2]. Since this report, the implication of PD-1 molecules in sepsis-induced immunosuppression has been largely demonstrated both in experimental and clinical studies.

In septic mice, administration of anti-PD1/anti-PD-L1 antibodies enhanced bacterial clearance by preventing lymphocyte depletion and alterations [3, 4]. In a two-hit model nicely mimicking the occurrence of nosocomial infection (sepsis + secondary fungal infection), those antibodies significantly improved survival [4]. In septic patients, several groups reported increased PD-1 expression on lymphocytes and monocytes [5–8]. Depending on studies, this overexpression was regularly observed to be associated with increased risk of nosocomial infections and mortality. Of note, other co-inhibitory receptors (BTLA, CTLA-4, TIM-3, LAG-3) are also overexpressed in sepsis. Along with altered functional responses (proliferation/IFN-γ production), this depicts a full picture of sepsis-induced lymphocyte exhaustion. Importantly, those results obtained in circulating blood cells were confirmed locally in organs (lung and spleen), illustrating this overexpression is a severe and profound mechanism [8]. The magnitude of this immunosuppression is also exemplified by patients’ difficulty to fight the primary bacterial infection, decreased resistance to secondary nosocomial infections and reactivation of viral infections [9]. Noteworthy, the implication of PD-1 molecules was recently reported in Ebola disease pathophysiology (which shares many clinical similarities with septic shock). While every Ebola-infected patient showed massive T-cell activation irrespective of outcome, the increased expression of these immune checkpoint molecules marked fatal evolution in some patients and correlated with high viraemia [10]. The authors hypothesized that these inhibitory molecules inhibit T-cell functions leading to poor viral clearance—a similar consequence to those observed during sepsis-induced immunosuppression.

Shao et al.’s study [1] agrees with this body of literature and provides two important novel aspects. Firstly, to our knowledge, this study represents the first description of a PD1 molecule, namely PD-L1, as an independent predictor of mortality in a multivariate analysis. Indeed, a combined score including the SAPS II score and PD-L1 expression provided good prognostic performances with an area under the curve in ROC analysis of 0.88. This illustrates the weight of initial severity and immunosuppression in sepsis. Secondly, it highlights the importance of monocytes in the PD-1 system and more broadly in sepsis-induced immune alterations. Indeed, the best result for predicting mortality is not obtained with PD-1-related molecule expressions on lymphocytes but rather with PD-L1 expression on monocytes. This again places monocytes at the centre of the pathophysiological game. Correlations between increased monocyte PD-L1 expression and decreased expression of HLA-DR or in-vitro TNF release—both typical markers of monocyte alterations—would thus have been interesting to support this hypothesis. In line with this, Shindo et al. [11] reported that anti-PD-1 antibody treatment had a positive effect on MHC class II molecule expression on macrophages and dendritic cells. Thus, the real importance of the PD-1 system on myeloid cells deserves further investigation.

Importantly, sepsis-induced impaired immune function is reversible and it remains possible to rejuvenate exhausted lymphocytes [12]. Among molecules able to participate in battling sepsis-induced immunosuppression, anti-PD-1/anti-PD-L1 antibodies thus appear as plausible candidates. In septic patients’ cells, ex-vivo blockade of the PD-1 system restored immune functions [13] while these agents currently provide excellent results in cancer therapy, in which they boost immune functions in adjunction to classical therapies. Nevertheless, this approach deserves to be envisaged with caution. Indeed, PD-1-related molecules are also involved in the tolerance phenomenon and belong to the normal process of T-cell differentiation/maturation and activation. In other words, PD-1 expression is not always a sign of T-cell alteration and does not necessarily mark reduced T-cell functions and exhaustion [14]. By unbalancing the immune system, immune checkpoint blockade thus favours the development of various autoimmune manifestations [15]. As a consequence, one important issue if we are to give anti-PD-L1 therapy to septic patients is to identify the right patient that could benefit from such therapy because it is unlikely, due to well-established heterogeneity of septic patients, that blocking a given immune checkpoint will be a magic bullet for all. Thus, robust standardized tools for patients’ stratification are highly desirable. In that sense, the work of Shao et al. [1] contributes to a better knowledge of PD-L1 expression as a potential biomarker.

## Abbreviations

PD-1, Programmed Death-1; PD-L1, Programmed Death-ligand 1

## References

[1] Shao R, Fang Y, Yu H, Zhao L, Jiang Z, Li CS (2016). Monocyte programmed death ligand-1 expression after 3-4 days of sepsis is associated with risk stratification and mortality in septic patients: a prospective cohort study. Crit Care.

[2] Huang X, Venet F, Wang YL, Lepape A, Yuan Z, Chen Y (2009). PD-1 expression by macrophages plays a pathologic role in altering microbial clearance and the innate inflammatory response to sepsis. Proc Natl Acad Sci U S A.

[3] Zhang Y, Zhou Y, Lou J, Li J, Bo L, Zhu K (2010). PD-L1 blockade improves survival in experimental sepsis by inhibiting lymphocyte apoptosis and reversing monocyte dysfunction. Crit Care.

[4] Chang KC, Burnham CA, Compton SM, Rasche DP, Mazuski R, Smcdonough J (2013). Blockade of the negative co-stimulatory molecules PD-1 and CTLA-4 improves survival in primary and secondary fungal sepsis. Crit Care.

[5] Zhang Y, Li J, Lou J, Zhou Y, Bo L, Zhu J (2011). Upregulation of programmed death-1 on T cells and programmed death ligand-1 on monocytes in septic shock patients. Crit Care.

[6] Guignant C, Lepape A, Huang X, Kherouf H, Denis L, Poitevin F (2011). Programmed death-1 levels correlate with increased mortality, nosocomial infection and immune dysfunctions in septic shock patients. Crit Care.

[7] Boomer JS, Shuherk-Shaffer J, Hotchkiss RS, Green JM (2012). A prospective analysis of lymphocyte phenotype and function over the course of acute sepsis. Crit Care.

[8] Boomer JS, To K, Chang KC, Takasu O, Osborne DF, Walton AH (2011). Immunosuppression in patients who die of sepsis and multiple organ failure. JAMA.

[9] Hotchkiss RS, Monneret G, Payen D (2013). Sepsis-induced immunosuppression: from cellular dysfunctions to immunotherapy. Nat Rev Immunol.

[10] Ruibal P, Oestereich L, Ludtke A, Becker-Ziaja B, Wozniak DM, Kerber R (2016). Unique human immune signature of Ebola virus disease in Guinea. Nature.

[11] Shindo Y, Unsinger J, Burnham CA, Green JM, Hotchkiss RS (2015). Interleukin-7 and anti-programmed cell death 1 antibody have differing effects to reverse sepsis-induced immunosuppression. Shock.

[12] Venet F, Foray AP, Villars-Mechin A, Malcus C, Poitevin-Later F, Lepape A (2012). IL-7 restores lymphocyte functions in septic patients. J Immunol.

[13] Chang K, Svabek C, Vazquez-Guillamet C, Sato B, Rasche D, Wilson S (2014). Targeting the programmed cell death 1: programmed cell death ligand 1 pathway reverses T cell exhaustion in patients with sepsis. Crit Care.

[14] Fuertes Marraco SA, Neubert NJ, Verdeil G, Speiser DE (2015). Inhibitory receptors beyond T cell exhaustion. Front Immunol.

[15] Michot JM, Bigenwald C, Champiat S, Collins M, Carbonnel F, Postel-Vinay S (2016). Immune-related adverse events with immune checkpoint blockade: a comprehensive review. Eur J Cancer.

